# Capillary Assembly of Anisotropic Particles at Cylindrical Fluid–Fluid Interfaces

**DOI:** 10.1021/acs.langmuir.3c00016

**Published:** 2023-04-18

**Authors:** Jack L. Eatson, Jacob R. Gordon, Piotr Cegielski, Anna L. Giesecke, Stephan Suckow, Anish Rao, Oscar F. Silvestre, Luis M. Liz-Marzán, Tommy S. Horozov, D. Martin A. Buzza

**Affiliations:** † Department of Physics & Mathematics, University of Hull, Hull HU6 7RX, U.K.; ‡ Department of Chemistry & Biochemistry, University of Hull, Hull HU6 7RX, U.K.; § AMO GmbH, Otto-Blumenthal-Str. 25, Aachen 52074, Germany; ∥ University of Duisburg-Essen, Bismarckstr. 81, Duisburg 47057, Germany; ⊥ Center for Cooperative Research in Biomaterials (CIC BiomaGUNE), Basque Research and Technology Alliance (BRTA), Paseo de Miramón 182, Donostia-San Sebastián 20014, Spain; # Centro de Investigación Biomédica en Red, Bioingeniería, Biomateriales y Nanomedicina (CIBER-BBN), Paseo de Miramón 182, Donostia-San Sebastián 20014, Spain

## Abstract

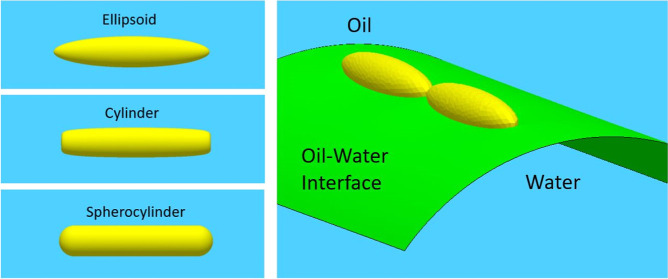

The unique behavior of colloids at liquid interfaces provides exciting opportunities for engineering the assembly of colloidal particles into functional materials. The deformable nature of fluid–fluid interfaces means that we can use the interfacial curvature, in addition to particle properties, to direct self-assembly. To this end, we use a finite element method (Surface Evolver) to study the self-assembly of rod-shaped particles adsorbed at a simple curved fluid–fluid interface formed by a sessile liquid drop with cylindrical geometry. Specifically, we study the self-assembly of single and multiple rods as a function of drop curvature and particle properties such as shape (ellipsoid, cylinder, and spherocylinder), contact angle, aspect ratio, and chemical heterogeneity (homogeneous and triblock patchy). We find that the curved interface allows us to effectively control the orientation of the rods, allowing us to achieve parallel, perpendicular, or novel obliquely orientations with respect to the cylindrical drop. In addition, by tuning particle properties to achieve parallel alignment of the rods, we show that the cylindrical drop geometry favors tip-to-tip assembly of the rods, not just for cylinders, but also for ellipsoids and triblock patchy rods. Finally, for triblock patchy rods with larger contact line undulations, we can achieve strong spatial confinement of the rods transverse to the cylindrical drop due to the capillary repulsion between the contact line undulations of the particle and the pinned contact lines of the sessile drop. Our capillary assembly method allows us to manipulate the configuration of single and multiple rod-like particles and therefore offers a facile strategy for organizing such particles into useful functional materials.

## 1 Introduction

Colloidal particles adsorbed at fluid–fluid interfaces are of great importance for a wide range of applications, including emulsification,^1,2^ encapsulation,^3^ food and pharmaceuticals,^4^ reconfigurable biomimetic systems,^5^ and surface patterning.^6,7^ They are also an ideal system for studying self-assembly due to a number of attractive features in this system. For example, because of the very high adsorption energies for particles with sizes greater than 10 nm,^8^ colloids at liquid interfaces are highly confined, allowing us to study self-assembly in two-dimensions.^9,10^ In addition, the fact that fluid–fluid interfaces are soft means that they can be easily deformed to generate strong and long-ranged capillary interactions between the particles, providing a powerful handle with which to control and harness self-assembly.^11−15^ A well-known example of capillary assembly is the so-called “Cheerios effect” which occurs when cereals in a bowl of milk spontaneously aggregate together.^16^ This effect is due to the deformation of the flat fluid–fluid interface by the gravitational force acting on the adsorbed particles so that, when the deformations from neighboring particles overlap, the particles experience a strong capillary attraction to reduce interfacial area and therefore aggregate together.

In the simple case where the adsorbed particles are spherical, the meniscus deformations due to gravity are circularly isotropic and the resultant capillary interactions are monopolar in nature.^17^ Such monopolar capillary interactions are generally negligible for spherical particles with sizes less than 10 μm.^2^ However, particles in this size range can still undergo significant capillary interactions if they are anisotropic, either in terms of particle shape or chemical heterogeneity. For example, when a chemically homogeneous rod-shaped particle such as an ellipsoid or a cylinder is adsorbed at a fluid–fluid interface, the constant contact angle requirement at the three-phase contact line around the particle cannot be satisfied by a flat interface for non-neutrally wetting rods and the meniscus spontaneously deforms to create quadrupolar deformations consisting of two rises and two falls in the contact line.^18−20^ These deformations lead to orientationally dependent quadrupolar capillary interactions,^19−21^ driving ellipsoidal particles to assemble side-to-side^21−23^ and cylindrical particles to assemble tip-to-tip.^20,23^ In the case of chemically heterogeneous particles such as Janus ellipsoids and cylinders (where the two “hemispheres” across the long axis of the particle have different surface chemistries), both shape and chemical anisotropy leads to more complex hexapolar contact line undulations and capillary interactions between the particles.^24−26^

The deformation of the fluid–fluid interface not only generates capillary interactions between adsorbed colloidal particles, but can also be used as an external field to direct self-assembly. For example, when rod-shaped particles are adsorbed at a curved interface, the rods will rotate until their quadrupolar rise axis is aligned with the principal axis of curvature where the interface is concave up.^27,28^ Indeed, Lewandowski et al. have shown that, when assembling two cylindrical particles at the curved interface, it was possible to suppress tip-to-tip assembly in favor of side-to-side assembly, by increasing the curvature of the interface so that the energy penalty for the cylinders to deviate from the orientation imposed by the host interface becomes too prohibitive.^28^ In addition, when rod-shaped particles are adsorbed at a fluid–fluid interface with a non-uniform curvature, the particles migrate toward regions of high curvature and simultaneously align themselves along the principal axis of curvature of the host interface.^29^

In this paper, we study the self-assembly of anisotropic rod-shaped particles at a cylindrical interface formed by a sessile liquid drop. Specifically, using the finite element method Surface Evolver,^30^ we study the assembly of single and multiple rods as a function of drop curvature and particle properties such as shape (ellipsoid, cylinder, and spherocylinder), contact angle, aspect ratio, and chemical heterogeneity (homogeneous and triblock patchy). The simple curved geometry of cylinders (constant finite curvature transverse to the cylinder and zero curvature along the cylinder) allows us to elucidate the interplay between interfacial curvature and particle properties in determining the configuration of single and multiple rods. Surprisingly, we find that, although the lateral dimension of the cylindrical drop is larger than the length of the rods in all cases studied, the curved interface allows us to effectively control the orientation of the rods so that they lie parallel, perpendicular, or oblique, with respect to the cylindrical drop. In addition, by tuning particle properties to achieve parallel alignment of the rods, we show that the cylindrical drop geometry favors tip-to-tip assembly for two rods, not just for cylinders, but also for ellipsoids and triblock patchy rods. Finally, although there are no curvature gradients in the host interface that can be used to control particle position, we can still achieve spatial confinement of the rods transverse to the cylindrical drop by using the capillary repulsion from the pinned contact lines of the sessile drop.^31,32^

## 2 Theoretical Model and Methods

In this section, we describe the geometry and thermodynamics of the composite system consisting of rod-like particles adsorbed at a sessile cylindrical liquid drop and the Surface Evolver method used to study this system theoretically.

**Figure 1:**
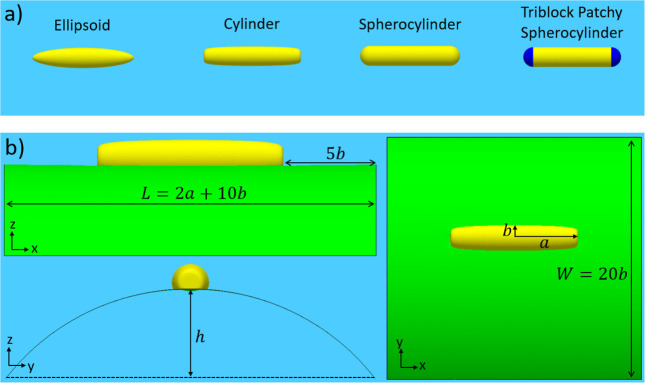
(a) Geometry of the simulated rod-like particles; (b) geometry of a rod-like particle adsorbed at a cylindrical sessile drop.

For rod-like particles, we consider three different particle shapes, namely, ellipsoids, cylinders, and spherocylinders (Figure 1). For ellipsoids and cylinders, we use the super-ellipsoid equation^23,33^
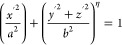
1to define the particle shape, where *x*^′^, *y*’, *z*’ are the Cartesian coordinates in the particle reference frame (see below in this section), *a*, *b* are the semi-major and semi-minor lengths of the rod, respectively, and η is a sharpness parameter that defines the sharpness of the super-ellipsoid edge. We use η = 1 for ellipsoids and η = 4 for cylinders (i.e., we consider cylinders with rounded edges; see Figure 1a). For spherocylinders, we use
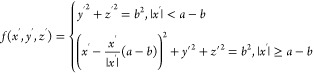
2Note that in all cases, the particle aspect ratio is *a*/*b*.

In terms of surface chemistry, we consider ellipsoids, cylinders, and spherocylinders with homogeneous surface chemistry, as well as spherocylinders with triblock patchy surface chemistry (Figure 1a).

For the cylindrical sessile drop, we consider a drop with a rectangular base of width *W* = 20*b* and length *L* = 2*a* + 10*b*. For convenience, we refer to the top and bottom fluid phases as oil and water, respectively (i.e., the fluid making up the drop is water), though our model is in fact general and applies to any fluid–fluid interface. Assuming the origin of the lab frame in Cartesian coordinates to be at the center of the base with *z* perpendicular to the base and *x*, *y* parallel and perpendicular to the long axis of the cylinder, respectively, we assume that the contact lines of the cylindrical drop at *y* = ±10*b* are pinned and apply reflecting boundary conditions for the interface at *x* = ±(*a* + 5*b*) (see Figure 1b). Note that the width of the drop is greater than the particle length for all cases studied in this work (*W* > 2*a*), and we have chosen *L* to be large enough so that for a particle positioned at the center of the drop (*x*, *y* = 0), the effect of the reflecting boundary conditions is small and the particle is effectively isolated. We fix the curvature of the cylindrical drop by applying a Laplace pressure of γ_ow_/*R* across the interface, where γ_ow_ is the oil–water interfacial tension and *R* is the radius of the cylinder in the absence of any adsorbed particles. Although the behavior of adsorbed rods is controlled by the curvature of the cylindrical drop,^27−29^ it is easier to control and measure the height of the drop experimentally. For convenience, we therefore parameterize the curvature of the drop using the drop height in the absence of adsorbed particles, *h*, which is related to *R* and *W* according to . Note that we consider drop heights *h* > *b*, so that the substrate does not play a critical role in determining particle behavior, that is, the adsorbed particles are in the flotation rather than the immersion regime.^11,12^

**Figure 2:**
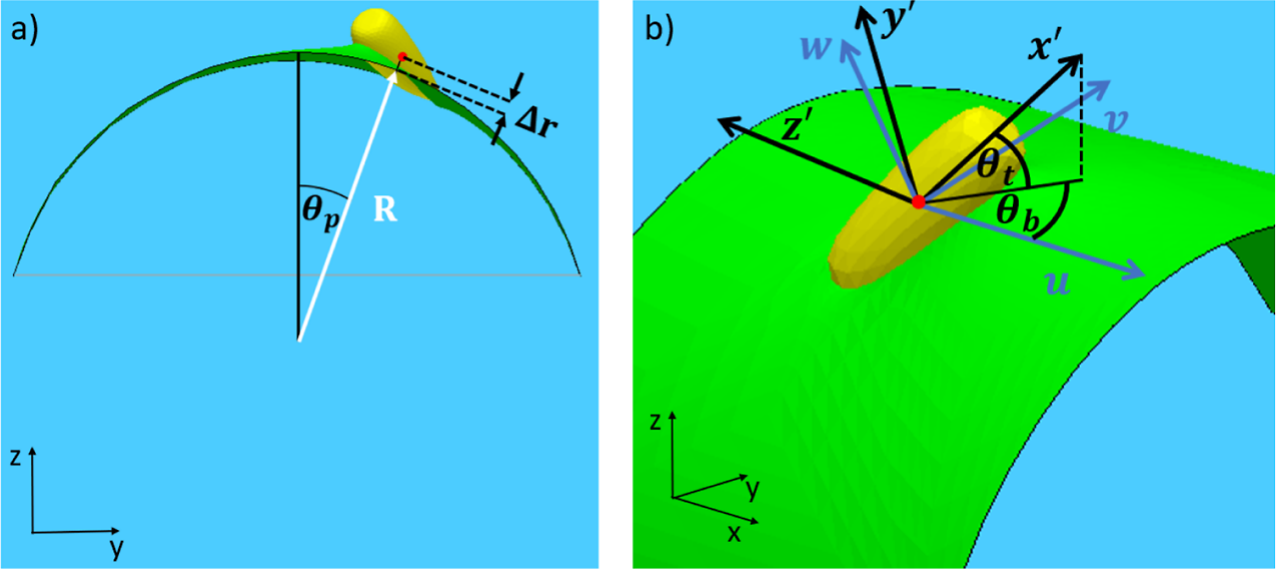
Degrees of freedom of a rod adsorbed at the cylindrical interface; the red dot represents the center of mass of the rod. (a) Cylindrical polar coordinates used to specify the position of the rod; (b) bond angle θ_b_ and tilt angle θ_t_ (defined with respect to the interfacial frame *u*, *v*, *w*) used to specify the orientation of the rod.

In order to specify the center-of-mass position of the adsorbed particle, we use cylindrical polar coordinates, where the position transverse to the cylindrical drop is given by the polar angle θ_p_, the radial distance from the long axis of the cylindrical drop is given by *r*_p_ = *R* + Δ*r*, and the position along the long axis is given by *x*_p_ (see Figure 2a).

In order to specify the orientation of the adsorbed particle, we first define a Cartesian coordinate system in the interfacial frame (*u*, *v*, *w*), with the origin coinciding with the particle center and *u*, *v*, *w* pointing in the directions of change for the cylindrical coordinates *x*, θ, *r*, respectively (see Figure 2b); the interfacial frame coordinates are related to the lab frame coordinates (*x*, *y*, *z*) by
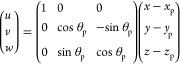
3where *y*_p_ = *r*_p_sinθ_p_, *z*_p_ = *r*_p_cosθ_p_ are the center-of-mass coordinates of the particle in the lab frame. Since we are considering axisymmetric rod-like particles, the orientation of the particle can be specified relative to (*u*, *v*, *w*) using two angles that we call the bond angle θ_b_ and the tilt angle θ_t_, as defined in Figure 2b.

Finally, we can define a Cartesian coordinate system in the particle frame , with *x*^′^ aligned along the semi-major axis of the particle and *y*^′^, *z*^′^ aligned along the semi-minor axes of the particle. These coordinates are related to the interfacial frame (*u*, *v*, *w*) coordinates by the following rotational transformations^34^
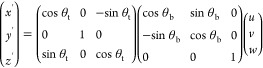
4

For micron or sub-micron particles, which is the focus of this work, gravity is negligible and the energy of the composite system is primarily due to the interfacial energy, which is given by^33,35^
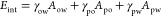
5where γ_ow_, γ_po_, and γ_pw_ are the interfacial tensions and *A*_ow_, *A*_po_, and *A*_pw_ are the areas of the oil–water, particle–oil, and particle–water interfaces, respectively. We have neglected line tension contributions in eq 5 because these are sub-dominant compared to interfacial tensions for the particles that we are considering, where *a*, *b* > 10 nm.^36^ We can simplify eq 5 by eliminating either the particle–water or the particle–oil interface from the problem. For example, using *A*_pw_ = *A* – *A*_po_ (where *A* is the total area of the particle) and Young’s equation γ_ow_cosθ_w_ = γ_po_ – γ_pw_ (where θ_w_ is the contact angle) and dropping irrelevant constant terms, we can eliminate the particle–water interface from eq 5 to obtain
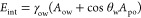
6

For a given particle configuration, the energy given by eq 6 (or its equivalent obtained by eliminating the particle-oil interface) is calculated using Surface Evolver.^30^ This is a finite element software that represents each interface as a mesh of triangles. The resultant vertices are then displaced to minimize the total interfacial energy, subject to the constraints of the boundary conditions of the simulation box, particle geometry, and the Laplace pressure.^30,33−35^ Note that the constant contact angle requirement around the particle is automatically satisfied within this scheme since Young’s equation arises from minimization of the total interfacial energy. We work with length and energy units such that *b* = 1 and γ_ow_ = 1, and we use a variable triangular mesh edge length between 0.02b and 0.1b and quadratic edges to capture the shape of the fluid–fluid interface and three-phase contact line more accurately. The Surface Evolver script that we used to generate the results in this paper is available as part of Supporting Information.

In this paper, we only consider particles with aspect ratio ≥2.5, where the equilibrium tilt angle at a flat interface is θ_t_ = 0^°^, that is, particles are in the “side-on” state.^33^ For the curved cylindrical interfaces and representative rod-shaped particles we are considering in this paper, we have checked that this result remains true, independent of the bond angle θ_b_; we have therefore set θ_t_ = 0^°^ in all simulations. In a typical simulation, we specify the position variables θ_p_, *x*_p_, and the bond angle θ_b_ of the rod-like particles, but allow the radial coordinate Δ*r* to equilibrate for a given particle configuration.

## 3 Results and Discussion

### 3.1 Single Rods at a Flat Interface

**Figure 3:**
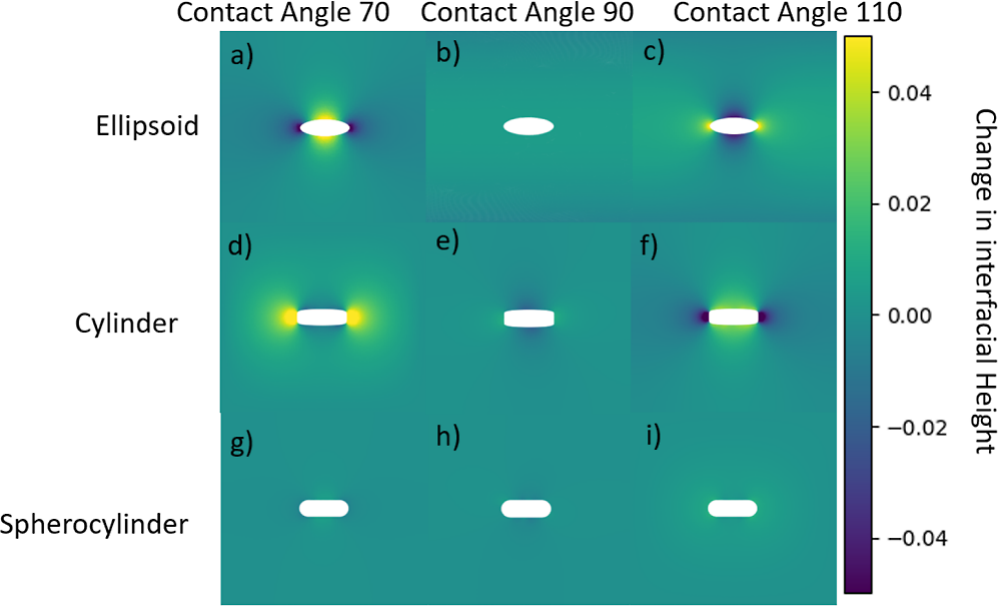
Contour plot of meniscus deformation around rod-like particles with aspect ratio 2.5 and homogeneous surface chemistry, adsorbed at a flat fluid–fluid interface for different particle shapes and contact angles.

In order to establish a baseline for our simulations of particles at cylindrical interfaces, we first analyze the behavior of single rods with homogenous surface chemistry at a flat interface (we consider the behavior of patchy rods in Section 3.5 below). In Figure 3 we show contour plots of the meniscus deformation for particles with aspect ratio *a*/*b* = 2.5, for different particle shapes (ellipsoids, cylinders and spherocylinders) and contact angles (θ_w_ = 70, 90, 110°). As expected, no meniscus deformations are observed for spherocylinders or neutrally wetting ellipsoids or cylinders (θ_w_ = 90^°^). On the other hand, for non-neutrally wetting ellipsoids and cylinders, we see quadrupolar meniscus deformations, in agreement with the literature.^18−20^ Good agreement with the literature is also obtained for the direction of the contact line curvature, relative to the long axis of the particle. For ellipsoids, the quadrupolar rise axis lies parallel and perpendicular to the long axis, for hydrophobic (θ_w_ = 110^°^) and hydrophilic (θ_w_ = 70^°^) particles, respectively. In contrast, for cylinders, the quadrupolar rise axis lies parallel and perpendicular to the long axis, for hydrophilic and hydrophobic particles, respectively. This means that the contact line curvature can be controlled by tuning particle shape and wettability, providing an effective way to control the orientation of rod-like particles at a cylindrical drop.

### 3.2 Single Rods at a Cylindrical Interface–Particle Orientation

Having studied the behavior of single rods with homogenous surface chemistry at a flat interface, we next analyze their behavior at a cylindrical interface. Specifically, in this section, we study the impact of particle shape, contact angle, aspect ratio, and droplet curvature on particle orientation, as specified by the bond angle θ_b_. As discussed in Section 2, since we are considering particles with aspect ratio ≥2.5, we set the tilt angle to θ_t_ = 0^°^.^33^ In addition, since we are interested in the effect of interfacial curvature on the orientation of isolated rods, we set θ_p_ = 0^°^, *x*_p_ = 0 (i.e., particle at apex of cylindrical drop, in the center of simulation box) to minimize the impact of the pinned contact line and reflecting boundary conditions of the cylindrical drop. We then calculate the energy of the system as a function of bond angle from θ_b_ = 0 to 90^°^ in increments of 1^°^, noting that the energy only needs to be calculated within this range due to the symmetry of the energy with respect to θ_b_.

**Figure 4:**
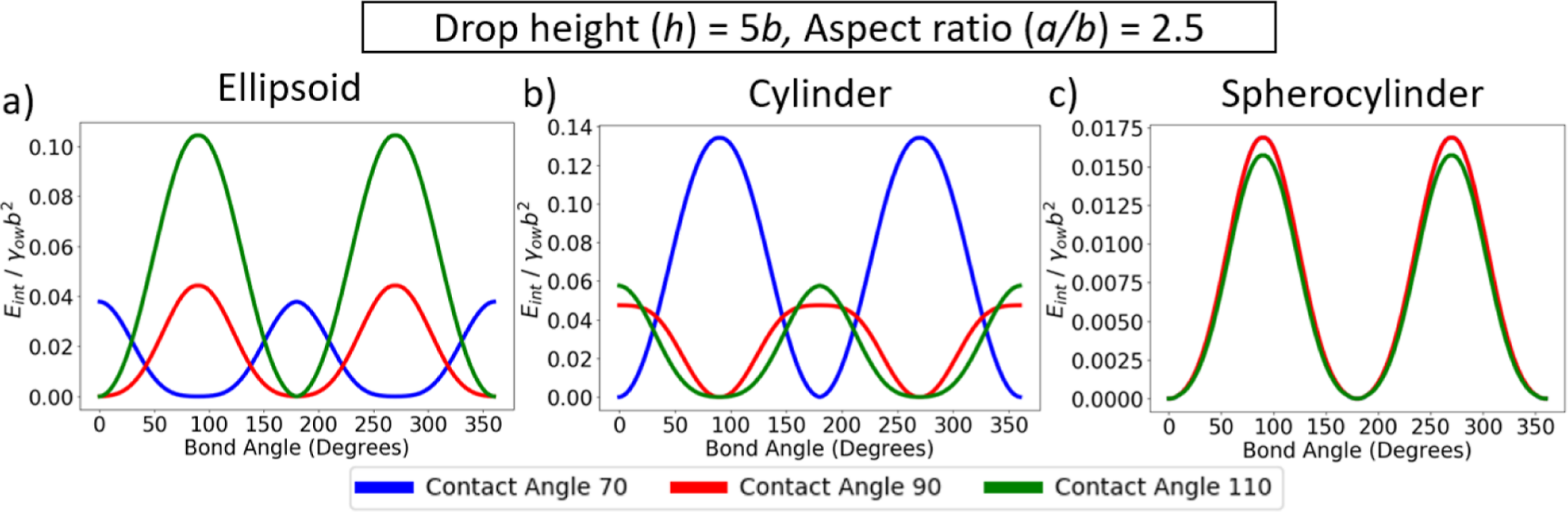
Interfacial energy as a function of bond angle for relatively short rods with different contact angles, adsorbed at a cylindrical interface, for (a) ellipsoids; (b) cylinders; and (c) spherocylinders. All rods have θ_p_ = 0^°^, *x*_p_ = 0, θ_t_ = 0^°^. Note that in (c), the curve for θ_w_ = 70^°^ is not visible as it lies underneath the curve for θ_w_ = 90^°^.

We first discuss the effect of contact angle on the orientation of rods with different shapes for relatively short rods (aspect ratio = 2.5) and a cylindrical drop height *h* = 5*b*. In Figure 4a, we plot interfacial energy (relative to the minimum energy state) as a function of bond angle θ_b_ for ellipsoids with contact angles θ_w_ = 70, 90, 110°. The equilibrium orientation of the ellipsoids (i.e., θ_b_ corresponding to the energy minimum) clearly depends on the contact angle: for hydrophilic ellipsoids (θ_w_ = 70^°^), the particles are aligned perpendicular to the cylindrical drop, that is, θ_b_ = 90^°^, whereas for hydrophobic ellipsoids (θ_w_ = 110^°^), the particles are aligned parallel to the cylindrical drop, that is, θ_b_ = 0^°^. This result can be readily understood from the fact that when particles with a capillary quadrupole are adsorbed at a curved interface, the particle will rotate to try to align its quadrupolar rise axis to the principal axis of curvature of the host interface (where the interface is concave up) to minimize the distortion to the host interface.^27−29^ Since hydrophilic and hydrophobic ellipsoids have their rise axis perpendicular and parallel to the long axis, respectively (see Figure 3), but the principal axis of curvature for a cylindrical drop is parallel to its long axis, it is not surprising that hydrophilic ellipsoids align perpendicular to the cylindrical drop whereas hydrophobic ellipsoids align parallel to it.

We additionally found (Figure 4a) that neutrally wetting ellipsoids (θ_w_ = 90^°^) preferentially align parallel to the cylindrical drop. Since these ellipsoids do not possess an intrinsic capillary quadrupole (see Figure 3), the primary driving force for such an alignment is not contact line curvature but shape anisotropy. Specifically, because of the curvature of the cylindrical interface, an anisotropic particle such as an ellipsoid removes a larger area of the energetically unfavorable oil–water interface when it is parallel, rather than perpendicular, to the cylindrical drop and therefore the parallel orientation has a lower energy. Shape anisotropy also has an impact on the orientational energy of the non-neutrally wetting ellipsoids (Figure 4a). For hydrophobic ellipsoids, where both contact line curvature and particle anisotropy favor parallel alignment, that is, where the two effects are synergetic, both the depth and curvature of the energy minima (the latter being proportional to the “spring constant” of the potential confining the rod to its equilibrium orientation) are greater compared to the case of hydrophilic ellipsoids, where contact line curvature favors perpendicular alignment but particle anisotropy favors parallel alignment, that is, where the two effects are antagonistic.

In Figure 4b, we plotted the interfacial energy as a function of bond angle for cylinders with contact angles θ_w_ = 70, 90, 110°. In this case, the dependence of particle orientation on the contact angle is opposite to that for ellipsoids, with hydrophilic and hydrophobic cylinders aligning parallel and perpendicular to the cylindrical drop, respectively. This difference is not surprising because the orientation of the quadrupolar rise axis relative to the long axis of the particle is opposite for cylinders compared to ellipsoids for a given contact angle (see Figure 3). However, similar to ellipsoids, the depth and curvature of the energy minima are greater for cylinders in the parallel orientation, compared to those in the perpendicular orientation, due to the synergistic effect of contact line curvature and shape anisotropy. Unexpectedly, we see that neutrally wetting cylinders preferentially align perpendicular to the cylindrical drop. We believe that this counterintuitive result is due to the short cylinders considered here (*a*/*b* = 2.5) removing a larger area from the oil–water interface in the perpendicular orientation than in the parallel orientation; as discussed in this section below, for longer cylinders, particle anisotropy always favors the parallel orientation. Note that in Figure 4, the energy scale for the orientational energy is slightly larger for cylinders compared to ellipsoids and we attribute this difference to the larger amplitude for contact line undulations in cylinders compared to ellipsoids. For example, for the particles shown in Figure 3, the difference between maximum and minimum height of the contact line is approximately 0.15*b* for cylinders and 0.11*b* for ellipsoids.

In Figure 4c, we plot interfacial energy as a function of bond angle for spherocylinders with contact angles θ_w_ = 70, 90, 110°. For all contact angles, spherocylinders are found to lie parallel to cylindrical drop. This is not surprising since spherocylinders do not possess an intrinsic capillary quadrupole (Figure 3) and particle orientation is therefore primarily determined by particle anisotropy, which favors the parallel orientation. We also note that the energy scale of the orientational energy for spherocylinders is almost one order of magnitude smaller than that for ellipsoids and cylinders. This effect is again due to spherocylinders not possessing an intrinsic capillary quadrupole, so that the only interfacial deformations are those induced by the curvature of the host interface, which are much weaker.

**Figure 5:**
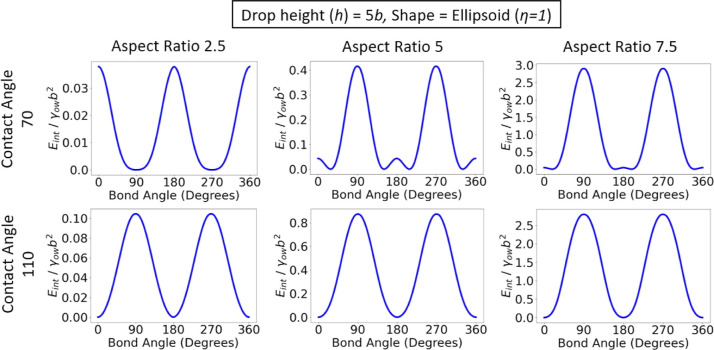
Interfacial energy as a function of bond angle for ellipsoids with different aspect ratios and contact angles, adsorbed at a cylindrical interface. All rods have θ_p_ = 0^°^, *x*_p_ = 0, and θ_t_ = 0^°^.

Next, we study the effect of aspect ratio on the orientation for particles of different shapes and contact angles. In Figure 5 we plot the orientational energies for ellipsoids for a cylindrical drop of height *h* = 5*b*. When the contact line curvature favors parallel alignment (θ_w_ = 110^°^), increasing the aspect ratio of the ellipsoids only leads to an increase in the energy scale of the potential well, but does not change the equilibrium orientation. This is as we would expect because the effect of particle anisotropy on particle orientation is synergistic to the effect of contact line curvature in this case.

However, when the contact line curvature favors perpendicular alignment (θ_w_ = 70^°^), particle anisotropy is antagonistic to contact line curvature. As the aspect ratio of the ellipsoid is increased from *a*/*b* = 2.5 to 5 and 7.5, particle anisotropy simultaneously leads to the formation of high energy barriers at θ_b_ = 90, 270^°^ and suppresses the energy barrier due to contact line curvature at θ_b_ = 0, 180, 360°. Intriguingly, the competition between particle anisotropy and contact line curvature means that the ellipsoid does not align either parallel or perpendicular in this case, but instead aligns obliquely to the long axis of the cylindrical drop, where the bond angle of the oblique orientation is determined by the length of the particle relative to the radius of curvature of the drop.

**Figure 6:**
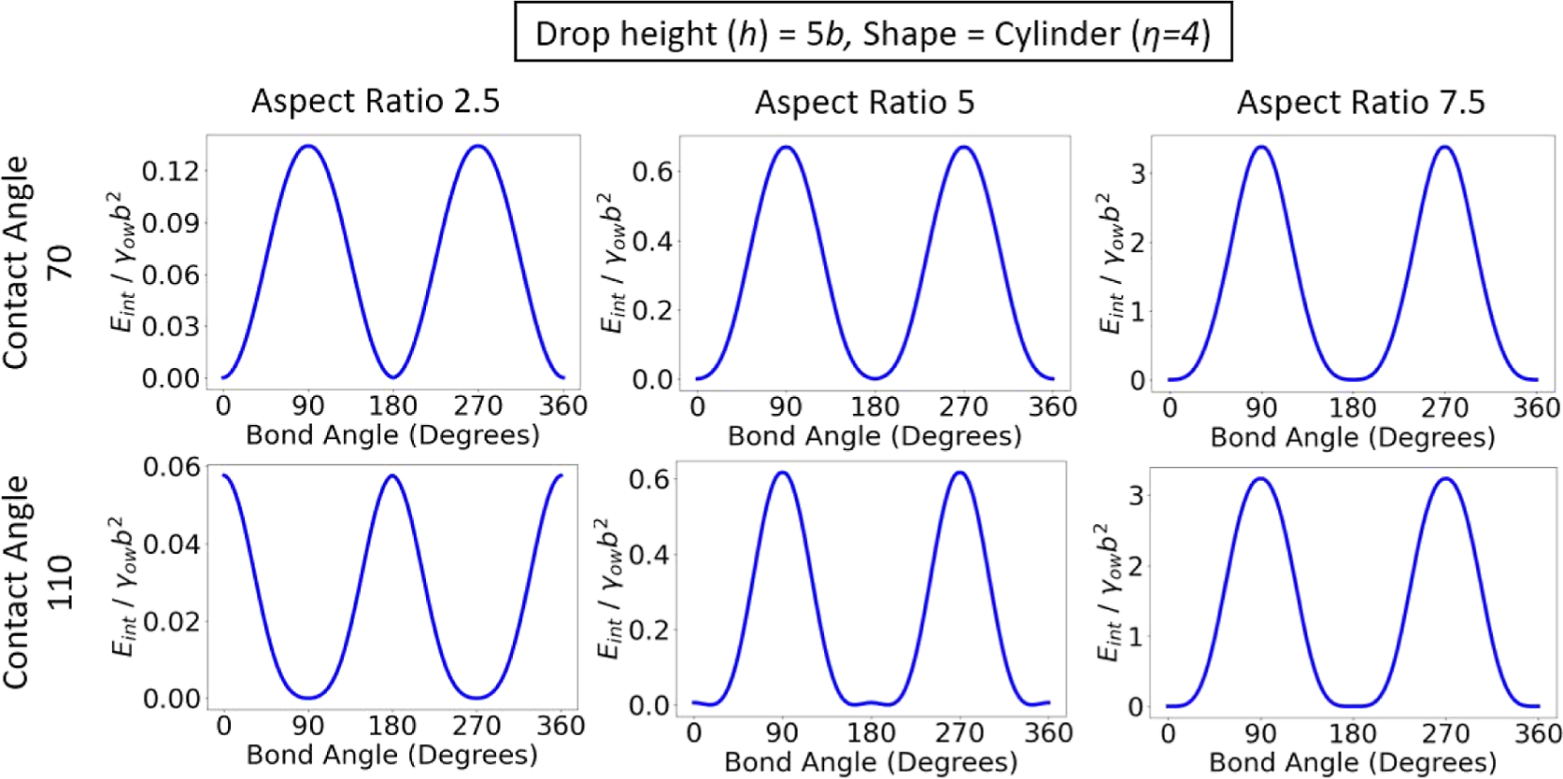
Interfacial energy as a function of bond angle for cylinders with different aspect ratios and contact angles, adsorbed at a cylindrical interface. All rods have θ_p_ = 0^°^, *x*_p_ = 0, and θ_t_ = 0^°^.

For cylindrical particles (Figure 6), we see the same trends as those for ellipsoids, except that the parallel orientation is now observed for θ_w_ = 70^°^ (where particle anisotropy and contact line curvature are synergistic), whereas the novel oblique orientation is observed for θ_w_ = 110^°^ (where particle anisotropy and contact line curvature are antagonistic). Note that the effect of particle anisotropy is stronger for cylinders compared to ellipsoids, so that the energy barrier at θ_b_ = 0, 180, 360° is strongly suppressed for *a*/*b* = 5 and essentially disappears for *a*/*b* = 7.5.

For spherocylinders, which do not have an intrinsic capillary quadrupole so that contact line curvature essentially plays no role in determining particle orientation, we find that the particles are in the parallel orientation for both θ_w_ = 70 and 110^°^ but the depth of the confining potential well increases with increasing particle aspect ratio (Figure S1).

It should be noted that the oblique orientations seen for ellipsoids and cylinders in Figures 5 and 6 only arise when the particle is long enough relative to the radius of curvature of the host interface. For example, in Figure 5, the oblique orientation is observed for rods with aspect ratios *a*/*b* = 5 and 7.5, where 2*a*/*R* = 0.8 and 1.2, respectively, but not for particles with aspect ratio 2.5, where 2*a*/*R* = 0.4. This explains why the oblique orientation was not observed in previous studies, for example, in Lewandowski et al.^28^ for cylindrical particles adsorbed at a plane–parabolic interface, where 2*a*/*R* = 6 × 10^–3^.

In Supporting Information, we also studied the effect of changing droplet curvature on the orientation of rod-shaped particles and find that increasing drop height (and hence interfacial curvature) at a fixed rod length (Figure S2) produces the same trend for the orientation of the rods as increasing rod length at a fixed interfacial curvature (Figures 5 and 6). This result can be readily understood by recognizing that the competition between particle anisotropy and contact line curvature depends primarily on the ratio between particle length and interfacial radius of curvature so that increasing particle length is essentially equivalent to decreasing the radius of curvature (i.e., increasing curvature). However, for the parameter ranges explored in this paper, we find that changing particle aspect ratio has a much bigger impact on orientational energy compared to changing droplet height (compare Figure S2 to Figure 5).

Note that even though the lateral dimension of the cylindrical drop is larger than the length of the rods in all cases studied above (*W* > 2*a*), the potential energy well confining the orientation of rods is very large. For example, for a hydrophobic ellipsoid with aspect ratio *a*/*b* = 5 and cylindrical height *h* = 5*b*, the potential energy well depth is  (Figure 5). For an oil-water interface with γ_ow_ = 30 mN/m, this translates into Δ*E*_int_ ≈ 6 × 10^6^ kT for a micron-sized rod with *b* = 1 μm and Δ*E*_int_ ≈ 600 kT for a nanorod with *b* = 10 nm. This result is consistent with what has been found by Lewandowski et al. who were able to control the orientation of a cylindrical microparticle using a curved interface, which has a radius of curvature much greater than the particle length.^28^

### 3.3 Single Rods at a Cylindrical Interface–Spatial Confinement

Having studied particle orientation in the previous section, in this section, we study the impact of the cylindrical interface on the spatial confinement of rod-like particles. Although no curvature gradients are present in a cylindrical sessile drop, which could provide control over particle position,^29^ spatial confinement of the rods transverse to the cylindrical drop can still be achieved due to steric repulsion from the substrate and capillary repulsion from the pinned contact lines.^31,32^ Both effects will be considered in this section. We restrict our analysis to rods in the parallel alignment as this is the most favorable alignment toward achieving the tip-to-tip assembly considered in the next section.

**Figure 7:**
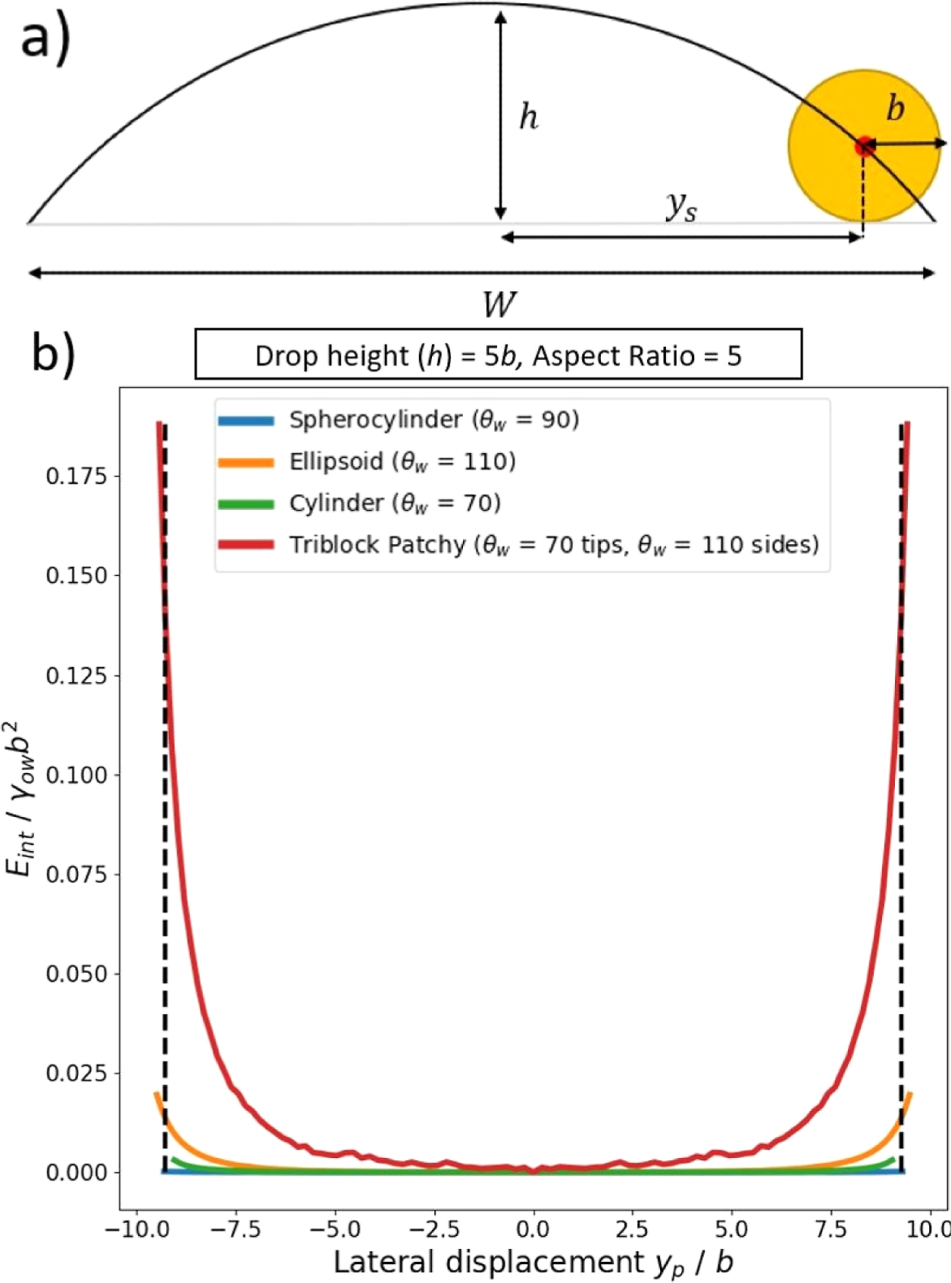
(a) Simplified model for calculating the maximum displacement of adsorbed rods lateral to a cylindrical drop, due to steric repulsion from the substrate. (b) Interfacial energy as a function of lateral displacement for adsorbed rods with different shapes and surface chemistry, which are aligned parallel to the cylindrical drop. The dashed black line indicates maximum lateral displacement allowed by steric repulsion with the substrate.

To estimate the spatial confinement due to steric repulsion from the substrate, we make the simplifying assumption that the fluid–fluid interface is the unperturbed cylindrical interface and that this interface goes through the center of the rod. In this case, from simple geometry, the maximum displacement of the rod from the origin in the *y* direction, *y*_s_, is given by (see Figure 7a)
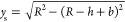
7

Substituting  into eq 7, we can rewrite eq 7 as
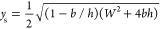
8

Note that in the limit where the short axis length of the rod *b* → 0, we recover *y*_s_ = *W*/2 as expected. From eq 8, we find that *y*_s_ decreases as we reduce the drop height *h*, that is, the rods become increasingly confined laterally, and indeed *y*_s_ = 0 for *h* = *b*. However, for the relatively large drop heights considered here, *y*_s_ is close to *W*/2, that is, the degree of lateral confinement due to steric repulsion from the substrate is insignificant. This point is illustrated in Figure 7b for the case *h* = 5*b*, where ±*y*_s_ is represented by the vertical dashed lines.

Next, we consider the lateral confinement due to capillary repulsion from the pinned contact line of the sessile drop. This repulsion arises because the meniscus around the particle would like to deform because of the particle’s contact line undulations, but this deformation is suppressed when the particle approaches the pinned contact line, causing the energy of the system to increase.^31,32^ To calculate this effect, we set θ_t_ = 0^°^, *x*_p_ = 0, and θ_b_ = 0^°^ and calculate the energy of the system as a function of the polar angle, from θ_p_ = 0^°^ to θ_max_, in increments of 1^°^, where θ_max_ is approximately the polar angle corresponding to *y* = *W*/2. Note that we only need to calculate the energy for positive θ_p_ because the energy is symmetric about θ_p_ = 0^°^.

In Figure 7b, we plot the energy of the system (relative to the energy at θ_p_ = 0^°^) as a function of *y*_p_ = *R*sinθ_p_, for ellipsoids with θ_w_ = 110^°^, cylinders with θ_w_ = 70^°^, and spherocylinders with θ_w_ = 90^°^, where the contact angles have been chosen to ensure that the rods are in the parallel orientation (triblock patchy rods will be discussed in Section 3.5 below). All particles have an aspect ratio of *a*/*b* = 5 and the drop height is *h* = 5*b*. We see that all particles are repelled by the pinned contact line at *y* = *W*/2. The repulsion is strongest for the ellipsoidal particle because this shape has the largest contact line undulation at the sides (see Figure 3). The repulsion is smaller for the cylindrical particle because the contact line undulation at the side is smaller, but the repulsion is weakest for spherocylinders, which do not have an intrinsic capillary quadrupole (see Figure 3).

However, our main conclusion from Figure 7b is that, for rod-shaped particles with homogeneous surface chemistry, the potential well around *y*_p_ = 0 is very flat, so that the lateral spatial confinement due to capillary repulsion from the pinned contact lines is weak. For example, for ellipsoids with *b* = 10 nm, the range of *y*_p_ values for which Δ*E*_int_ < 3 kT is −8.4*b* ≤ *y*_p_ ≤ 8.4*b* (3 kT is a reasonable estimate for the point where the confining potential starts to become significant compared to thermal energy), which corresponds to 91% of 2*y*_s_, the maximum lateral spatial range available to the ellipsoid due to steric repulsion by the substrate (i.e., the range between the dashed vertical lines in Figure 7). The spatial confinement for cylinders and spherocylinders with *b* = 10 nm is even weaker, with Δ*E*_int_ < 3 kT at *y*_p_ = ±*y*_s_. We therefore conclude that the lateral spatial confinement due to capillary repulsion from the pinned contact lines of the sessile drop is weak for rods with homogeneous surface chemistry.

### 3.4 Capillary Interaction and Self-Assembly of Rods at a Cylindrical Interface

Having considered the orientation and spatial confinement of single rods at a cylindrical interface in previous sections, we now consider the interaction and self-assembly of two rods at a cylindrical interface. We restrict our analysis to rods with parallel alignment and primarily consider the tip-to-tip assembly of such rods. However, we point out that it is also possible to use our method to orient the rods perpendicular to the cylindrical drop, which would favor side-to-side assembly.

For the sake of simplicity, we first consider the case where the long axes of the two parallel rods are aligned to each other, and they are both at the apex of the cylindrical drop, that is, θ_b_, θ_p_ = 0^°^; later in this section, we will consider the case where the rods are not aligned to each other. We also exploit the fact that the energy of one rod approaching the reflecting boundary of the simulation cell is equal to half the energy of a two-particle system where both rods are approaching each other. When calculating the tip-to-tip interaction between two rods, we therefore just need to consider a one-rod simulation where we vary the distance of the rod to the reflecting boundary. Note that when two rods in the tip-to-tip orientation are close to each other, they may induce a capillary dipole in each other, so that θ_t_ is no longer zero. To check the size of this effect, we performed Surface Evolver simulations of two ellipsoids in a mirror symmetric configuration at the smallest surface-to-surface separation we studied and calculated the interfacial energy of the system as a function of the tilt angle of the rods θ_t_. We found that the equilibrium tilt angle θ_t_ < 1^°^, suggesting that this capillary polarization effect is very small. We therefore set θ_t_ = 0^°^ for both interacting rods in our calculations.

**Figure 8:**
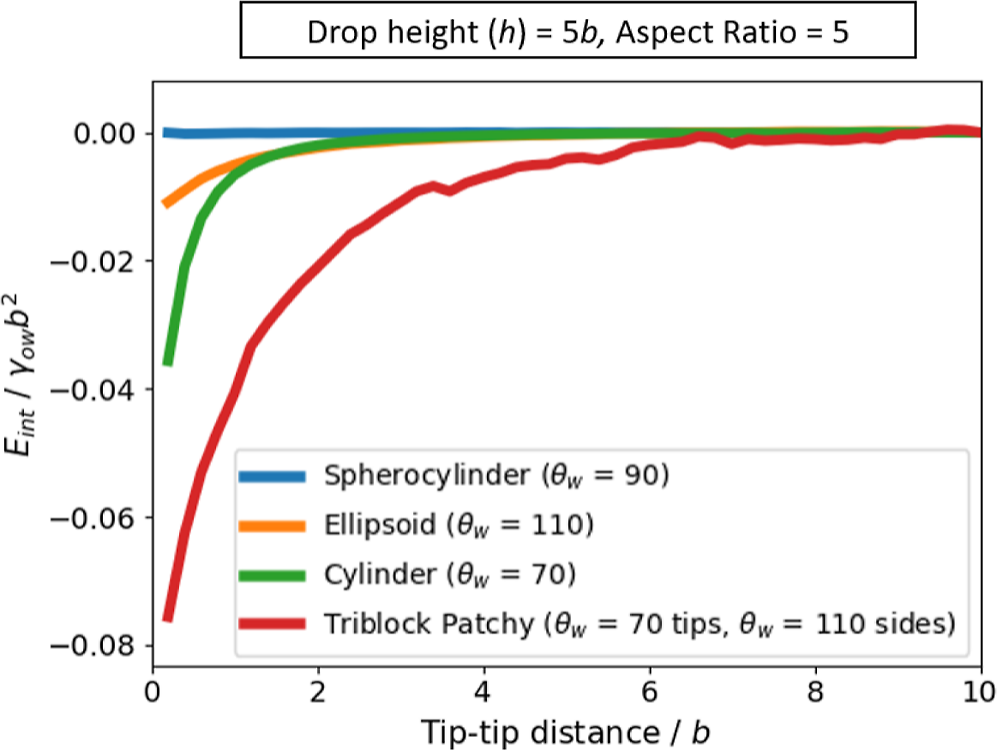
Tip-to-tip capillary interaction for adsorbed rods with different shapes and surface chemistry, which are aligned parallel to the cylindrical drop.

In Figure 8, we plot the tip-to-tip interaction energy between two rods (i.e., energy relative to the energy of the two rods at maximum separation) as a function of the surface-to-surface separation between the two rods, for ellipsoids with θ_w_ = 110^°^, cylinders with θ_w_ = 70^°^, and spherocylinders with θ_w_ = 90^°^ (triblock patchy rods will be discussed in Section 3.5 below). The contact angles have been chosen, so that all rod shapes are in the parallel alignment since, as explained at the start of this section, we are primarily interested in the tip-to-tip assembly of rods. All particles have an aspect ratio of *a*/*b* = 5 and the drop height is *h* = 5*b*. We observe that tip-to-tip attraction is strongest for cylindrical particles and weakest for spherocylinders. These results are similar to what has been observed at flat interfaces^23^ and is due to cylinders having the largest contact line undulation around their tips, compared to ellipsoids and spherocylinders (see Figure 3). Specifically, for nanoscale rods with *b* = 10 nm, at an oil-water interface with γ_ow_ = 30 × 10^–3^ N/m, the interaction energy for the tip-to-tip contact is 25 kT for cylinders, 8 kT for ellipsoids, and 0.05 kT for spherocylinders. The capillary interaction for nanoscale cylinders and ellipsoids is therefore significant, whereas that for spherocylinders is negligible compared to thermal energy.

Note that, at a flat interface, ellipsoids tend to approach each other tip-to-tip initially and then “roll-over” into the side-to-side configuration because of its lower energy.^19,23,37^ However, for ellipsoids at a cylindrical interface, the reduction in capillary interaction energy when going from the tip-to-tip to the side-to-side configuration is generally much smaller than the increase in orientational energy incurred in making this transition. For example, for ellipsoids with an aspect ratio *a*/*b* = 3 and contact angle θ_w_ = 80 or 100^°^, studied in Botto et al.,^23^ the reduction in capillary interaction energy is ≈ 0.001γ_ow_*b*^2^, whereas the increase in the orientational energy for the similar ellipsoid at a cylindrical interface in Figure 5 (*a*/*b* = 2.5, θ_w_ = 110^°^) is ≈ 0.1γ_ow_*b*^2^. Therefore, for two ellipsoids aligned tip-to-tip at a cylindrical interface of high enough curvature, the roll-over into the side-to-side configuration is suppressed and the ellipsoids remain assembled tip-to-tip. A similar phenomenon has also been observed in Lewandowski et al.^28^ for cylinders where for high enough curvatures of the host interface, the cylinders assemble side-to-side because the reorientation of the cylinders that would allow them to assemble tip-to-tip is suppressed. In the Supporting Information, we estimate the minimum cylindrical drop height (and hence minimum curvature of the host interface) required to suppress the roll-over transition and find that the minimum height is much smaller than *b* for typical rods (Figure S3). Since we are interested in the flotation regime (i.e., *h* > *b*), we can safely neglect the roll-over transition when studying the self-assembly of rod-like particles at a cylindrical interface.

Up to now, we have considered the capillary interaction between two parallel rods, which are already aligned tip-to-tip. However, from our previous discussion, we know that rods with homogeneous surface chemistry are only weakly confined transverse to the cylindrical drop. Therefore, the most usual case is in fact where the two interacting rods are not aligned initially. We now consider this more general case. Since the energy scale for rotating the rods away from their preferred parallel alignment is so high, we set θ_t_, θ_b_ = 0^°^ for both particles. However, even with this restriction on particle configuration, a full analysis of the two-particle problem is very expensive in Surface Evolver as it would require us to calculate the capillary interaction for different lateral positions of one of the rods and different positions of the second rod relative to the first. In order to make the problem numerically tractable, we therefore fix the position of the first rod and study the trajectory of the second rod at different starting positions relative to the first one. Specifically, to obtain an upper bound estimate for the effect of non-alignment on self-assembly, we fix the position of the first rod to be close to the edge of the cylindrical drop (roughly one rod diameter away, along the interface, from the pinned contact line of the cylindrical drop).

**Figure 9:**
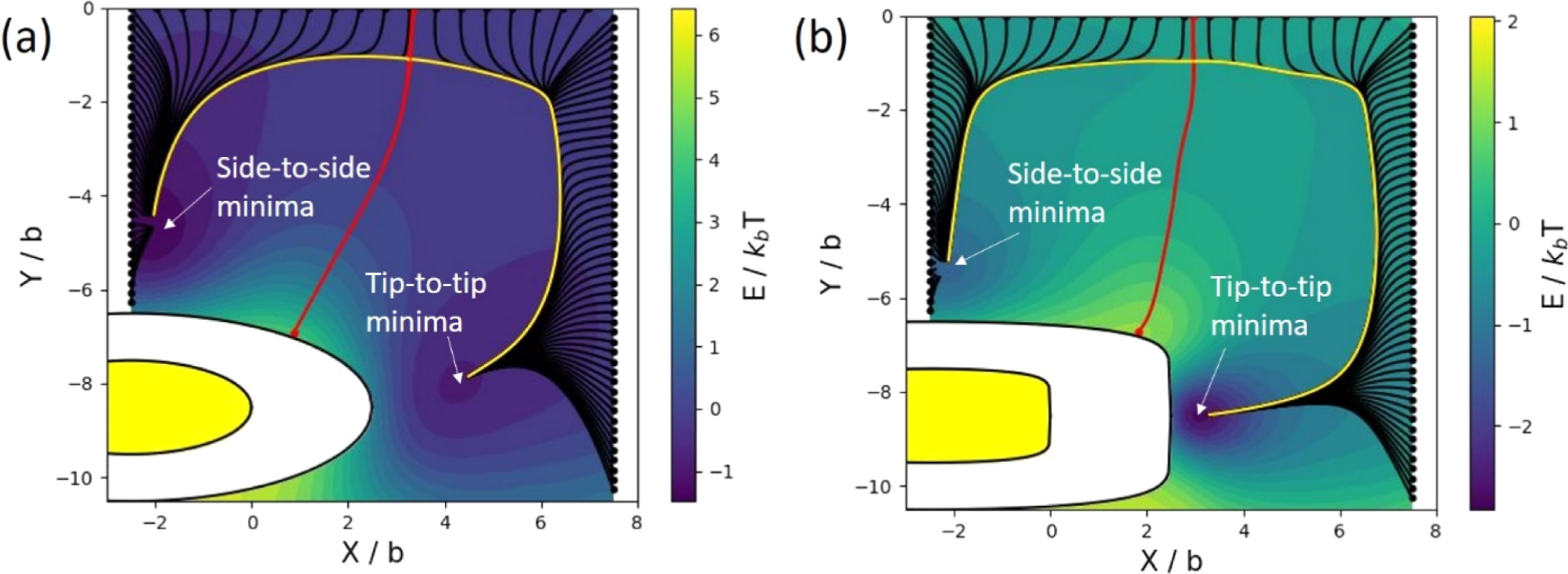
Capillary interaction energy of a two-particle system as a function of the position of the second particle relative to the first one, which is positioned at the edge of the cylindrical drop. The black lines are the trajectories of the second particle for different starting positions, the red curve is the separatrix that separates trajectories ending up in the tip-to-tip or side-to-side configurations, and the yellow lines are the dynamical attractors to which the trajectories converge at the later stages of their evolution. The energy landscape and trajectories are shown for (a) ellipsoids with *a*/*b* = 2.5, θ_w_ = 110^°^; (b) cylinders with *a*/*b* = 2.5, θ_w_ = 70^°^. The shaded-out white region on the bottom left of each plot represents the region excluded to the second particle due to steric repulsion with the first particle (colored yellow).

In Figure 9a,b, we plot the interaction energy of the two-particle system (i.e., energy relative to the energy of the two rods at maximum separation) as a function of the position of the second particle, expressed in terms of the coordinates *X* = *x*_p_,*Y* = *R*θ_p_, for ellipsoids with *a*/*b* = 2.5, θ_w_ = 110^°^ (Figure 9a) and cylinders with *a*/*b* = 2.5, θ_w_ = 70^°^ (Figure 9b) on a cylindrical drop with height *h* = 5*b*. The shaded-out region on the bottom left of each plot represents the region excluded to the second particle due to steric repulsion with the first one. Note that the energy landscape in Figure 9 was obtained by first calculating the energy for *X*, *Y* values on an approximately 30 × 30 grid. Since the capillary interaction between the rods is small, the resultant discrete energy landscape was quite noisy. Therefore, in order to obtain the full energy landscape for any *X*, *Y*, we use a radial basis function to interpolate the data with the Python package SciPy. Using the radial basis function allows us to use a smoothing parameter that makes the interpolation smoother by not insisting that it fits each discrete data point exactly. This procedure resulted in a smoother energy landscape, which we could use to calculate the trajectory of the second particle.

Assuming that the trajectory of the second particle follows paths of steepest descent in the interaction energy landscape,^38,39^ we plot the trajectories of the second particle for different initial positions on the edge of a rectangular region around the first one (black lines in Figure 9a,b). We see that, for initial positions closer to the tip of the first particle, the final state of the system is the tip-to-tip configuration, whereas for initial positions closer to the side of the first particle, the final state of the system is the side-to-side configuration. The red line shown in Figure 9a,b is the “separatrix” which demarcates the boundary between two different types of trajectories;^39,40^ all trajectories originating from points to the left of the separatrix will flow toward the side-to-side configuration but all trajectories originating from points to the right of the separatrix will flow toward the tip-to-tip configuration. Interestingly, trajectories on either side of the separatrix each converge to their own “dynamical attractor” in the later stages of their evolution (yellow lines), a feature that is commonly seen in many dynamical systems.^38−40^

Since the adsorption of rods onto the cylindrical drop is essentially a random process, the probability that an adsorbed rod will end up in the side-to-side or tip-to-tip configuration relative to a fixed rod at the edge of the cylindrical drop is proportional to the areas of the “domain of influence” of the fixed rod that are on either side of the separatrix (we define the domain of influence as the region where the capillary interaction energy ≳kT). In the far-field, we can approximate the capillary interaction energy between two rods with center-to-center separation *r* as ,^19−21^ where *H* is the amplitude of the contact line undulation and *r*_c_ ≈ (*a* + *b*)/2 is the average radius of the contact line. It is useful to estimate the radius of the rod *b* where the domain of influence of the fixed rod extends across the entire width of the cylindrical drop, that is, where . For a typical rod-like particle, for example, an ellipsoid with aspect ratio *a*/*b* = 2.5 and contact angle θ_w_ = 110^°^ where *H* ≈ 0.05*b* (see Figure 3), we find this radius to be *b* ≈ 50 nm. This means that for rods with *b* ≳ 50 nm, the domain of influence of the fixed rod extends beyond the width of the cylindrical drop, and the drop width acts as a cut-off length limiting the area to the left of the separatrix. In contrast, there is essentially no such cut-off along the length of the cylindrical drop and the area to the right of the separatrix therefore increases indefinitely as we increase *b*. These results show that for rods with *b* ≳ 50 nm, the cylindrical drop geometry favors tip-to-tip assembly for both ellipsoids and cylinders, even when the long axes of the two interacting rods are not lined up initially. Finally, if more than two rods are present at the cylindrical interface, our analysis suggests that capillary interaction will lead to the formation of a long chains of rods that are connected to each other tip-to-tip.

### 3.5 Triblock Patchy Rods

Up to now, we have considered rod-like particles with homogeneous surface chemistry and controlled contact line undulations through particle shape. In this section, we consider the assembly of patchy rods, which have heterogeneous surface chemistry. In order to minimize the effect of particle shape on self-assembly, we consider patchy spherocylinders. As discussed in the introduction, diblock patchy rods (i.e., Janus rods) have hexapolar contact line undulations.^24−26^ In order to compare the results in this section with those in the previous sections on particles with capillary quadrupoles, we therefore consider spherocylinders with triblock patchy geometry as this is the simplest patchy-particle geometry that possesses a capillary quadrupole; with advances in synthetic chemistry, such patchy particles can now be readily synthesized.^41^ In order to achieve parallel alignment of the rods, we consider spherocylinders with hydrophilic hemispherical caps (θ_w_ = 70^°^) and hydrophobic cylindrical sides (θ_w_ = 110^°^), so that the contact line is concave upward along the long axis of the particle (Figure 1a).

**Figure 10:**
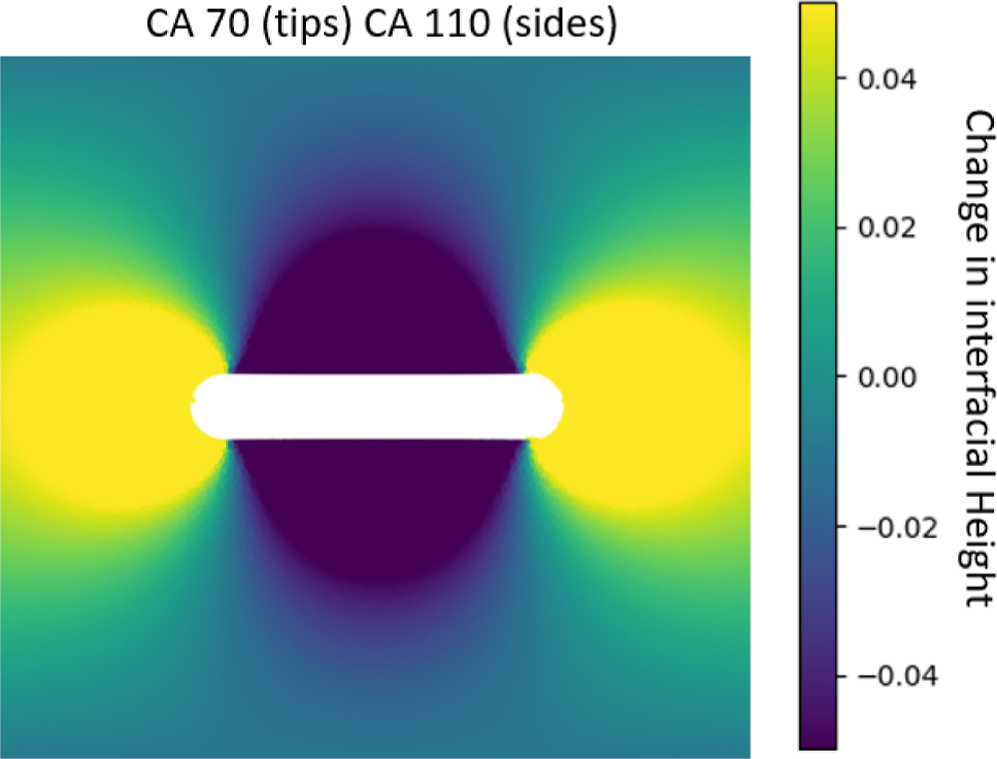
Contour plot of meniscus deformation around a triblock patchy particle with aspect ratio 5 adsorbed at a flat fluid–fluid interface.

In Figure 10, we show a contour plot of the meniscus deformation around the patchy particles at a flat plane. We can see that despite the spherocylinder shape, the triblock patchy particle has much larger contact line undulations both at the sides and the tips compared to rods with homogeneous surface chemistry (compare the range of interfacial heights in Figures 3 and 10). We anticipate that these large undulations will lead to much stronger spatial confinement and the tip-to-tip capillary interactions for the patchy rod compared to the non-patchy rods.

In Figure 7b, we plot the energy as a function of *y*_p_ = *R*sinθ_p_ for the triblock patchy rods with aspect ratio *a*/*b* = 5 at a cylindrical drop with height *h* = 5*b* compared to the corresponding non-patchy rods. As anticipated, the patchy rod experiences a much stronger spatial confinement lateral to the cylindrical drop. For example, the potential well depth is Δ*E*_int_ ≈ 0.14γ_ow_*b*^2^ for the patchy rod but only Δ*E*_int_ ≈ 0.02γ_ow_*b*^2^ for non-patchy ellipsoids; for γ_ow_ = 30 × 10^–3^ N/m, these well depths correspond to 100 and 15 kT, respectively, for nanoscale particles with *b* = 10 nm.

In Figure 8, we plot the tip-to-tip interaction energy as a function of separation between two patchy rods with aspect ratio *a*/*b* = 5 at a cylindrical drop with height *h* = 5*b* compared to the corresponding non-patchy rods. Once again, as anticipated, the capillary interaction is much greater for patchy rods compared to the non-patchy rods. For example, the interaction energy at contact is −0.08γ_ow_*b*^2^ for the patchy spherocylinder but only −0.04γ_ow_*b*^2^ for non-patchy cylinders; for γ_*ow*_ = 30 × 10^–3^ N/m, these contact energies correspond to −50 kT and −25 kT, respectively, for nanoscale particles with *b* = 10 nm. In addition, the range of the interactions is much greater for the patchy rod (≈6*b*) compared to the non-patchy rods (≈2*b* for the non-patchy cylinder). Since both lateral spatial confinement and tip-to-tip attraction are much greater for patchy rods, we conclude that tip-to-tip assembly at a cylindrical interface is much more likely for triblock patchy rods compared to non-patchy rods.

## 4 Conclusions

We have used the finite element method Surface Evolver to study the capillary assembly of rod-shaped particles adsorbed at a sessile liquid drop with cylindrical geometry. We considered the flotation regime where the drop height is greater than the diameter of the rods and studied the assembly of rods as a function of interfacial curvature, particle shape (ellipsoid, cylinder, and spherocylinder), contact angle, aspect ratio, and chemical heterogeneity (homogeneous and triblock patchy).

For rods with homogeneous surface chemistry, we can achieve very strong localization of particle orientation using cylindrical drops with a lateral width much greater than the length of the rods. By changing particle shape, contact angle, and aspect ratio, we can tune the interplay between interfacial curvature, particle contact line curvature, and particle anisotropy and control the rod not only to align parallel or perpendicular to the long axis of the cylindrical drop but also to align in novel oblique orientations. In contrast, we can only achieve weak spatial confinement of the rods transverse to the cylindrical drop because of the weak repulsion between the capillary quadrupole of the particle and the pinned contact lines of the sessile drop.

For ellipsoids and cylinders oriented parallel to the cylindrical drop, the capillary interaction is strong when the rods are oriented tip-to-tip, even at the nanoscale. In contrast, the capillary interaction between spherocylinders in the parallel orientation is extremely weak because these particles do not possess an intrinsic capillary quadrupole. Since in the confined geometry of the cylindrical drop, rods in the parallel orientation are more likely to approach each other in a tip-to-tip orientation, whereas interfacial curvature suppresses the transition from the tip-to-tip to the side-to-side configuration, the cylindrical drop favors the tip-to-tip assembly of rod in the parallel orientation, not only for cylinders but also for ellipsoids.

Finally, for triblock patchy rods, which possess much larger contact line undulations compared to non-patchy rods, the stronger capillary quadrupole leads to stronger lateral spatial confinement and tip-to-tip capillary attraction, resulting in an even stronger tendency for patchy rods in the parallel orientation at a cylindrical interface to assemble tip-to-tip compared to non-patchy rods.

The proposed capillary assembly mechanism allows us to manipulate the configuration of single and multiple rod-like particles and therefore offers a facile strategy for organizing such particles into useful functional materials.

## Supporting Information Available

Orientational energy landscape for spherocylinders; orientational energy landscape for ellipsoids for different droplet heights; and energy barrier for the roll-over transition for ellipsoids (PDF)
Surface Evolver script used for this paper (TXT)
