# Supplementary material for: Capillary Assembly of Anisotropic Particles at Cylindrical Fluid–Fluid Interfaces
Source: Langmuir. 2023 Apr 18;39(17):6006–17. doi: 10.1021/acs.langmuir.3c00016 (PMC10157885; doi:10.1021/acs.langmuir.3c00016)
Supplement: Supplementary file 1 — la3c00016_si_001.pdf [file la3c00016_si_001.pdf]

Supplementary Information - Capillary assembly of anisotropic particles at cylindrical fluid-fluid interfaces

Jack L. Eatson[1], Jacob R. Gordon[2], Piotr Cegielski[3], Anna L. Giesecke[3,4], Stephan Suckow[3], Anish Rao[5], Oscar F. Silvestre[5,6], Luis M. Liz-Marzán[5], Tommy S. Horozov[2], D. Martin A. Buzza[1,*]

[1]*Department of Physics & Mathematics, University of Hull, Hull HU6 7RX, United Kingdom*
[2]*Department of Chemistry & Biochemistry, University of Hull, Hull HU6 7RX, United Kingdom*
[3]*AMO GmbH, Otto-Blumenthal-Str. 25, 52074 Aachen, Germany*
[4]*University of Duisburg-Essen, Bismarckstr. 81, 47057 Duisburg, Germany*
[5]*Center for Cooperative Research in Biomaterials (CIC biomaGUNE), Basque Research and Technology Alliance (BRTA), Paseo de Miramón 182, 20014 Donostia-San Sebastián, Spain.*
[6]*Centro de Investigación Biomédica en Red, Bioingeniería, Biomateriales y Nanomedicina (CIBER-BBN), Paseo de Miramón 182, 20014 Donostia-San Sebastián, Spain.*

We first study the impact of particle shape, contact angle, aspect ratio and droplet curvature on particle orientation for additional cases that complement the results presented in the main paper. As discussed in Section 2 of the main paper, since we are considering particles with aspect ratio ≥ 2.5, we set the tilt angle to $\theta_t = 0^\circ$. In addition, since we are interested in the effect of interfacial curvature on the orientation of isolated rods, we set $\theta_p = 0^\circ$, $x_p = 0$ (i.e., particle at apex of cylindrical drop, in the centre of simulation box) to minimise the impact of the pinned contact line and reflecting boundary conditions of the cylindrical drop.

In Figure S1 we plot interfacial energy as a function of bond angle θ_b for spherocylinders adsorbed at a cylindrical drop of height $h = 5b$ for contact angles $\theta_w = 70°$ (top row) and $\theta_w = 110°$ (bottom row) and aspect ratios $a/b = 2.5, 5, 7.5$. For spherocylinders which do not have an intrinsic capillary quadrupole so that contact line curvature essentially plays no role in determining particle orientation, we note that the particles are in the parallel orientation for both $\theta_w = 70°$ and $110°$, but the depth of the potential well confining particle orientation increases with increasing particle aspect ratio.

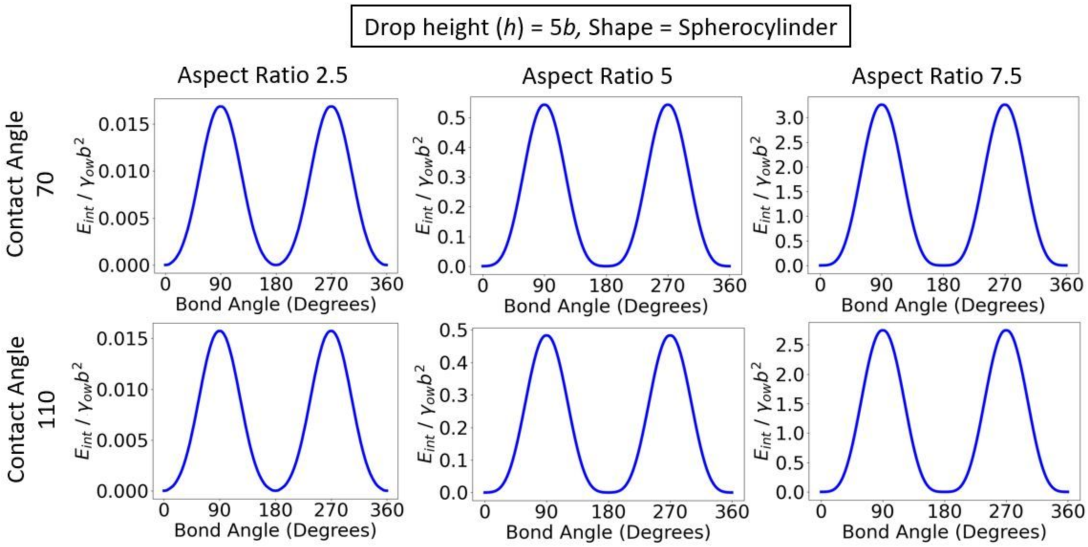

Figure S1. Interfacial energy as a function of bond angle for spherocylinders with different aspect ratios and contact angles, adsorbed at a cylindrical interface. All rods have $\theta_p = 0°$, $x_p = 0$, $\theta_t = 0°$.

Next, we study the effect of changing droplet curvature (parameterised by droplet height at fixed $W = 20b$) on the orientation of rod-shaped particles. For simplicity, we only present results for ellipsoids, but the same trends were observed for cylinders (results not shown). In Figure S2, we show the orientational energy for ellipsoids with aspect ratio $a/b = 2.5$ and contact angles $\theta_w = 70°$ (top row) and $\theta_w = 110°$ (bottom row) for cylinder drop heights $h = 2.5b$,$5b$, $7.5b$. For $\theta_w = 110°$, where parallel alignment of the ellipsoids is favoured (particle anisotropy and contact line curvature are synergistic), increasing interfacial curvature by increasing h leads to an increase in the depth of the potential well, but otherwise does not change the equilibrium orientation of the ellipsoid. On the other hand, for $\theta_w = 70°$, where perpendicular alignment of the ellipsoids is favoured (particle anisotropy and contact line curvature are antagonistic), a similar increase in the interfacial curvature causes an initial flattening of the minima at $\theta_b = 90°, 270°$ (for $h = 5b$), followed by emergence of a small energy barrier (for $h = 7.5b$), resulting in the novel oblique orientation of the ellipsoid that was also observed for hydrophilic ellipsoids in Figure 5.

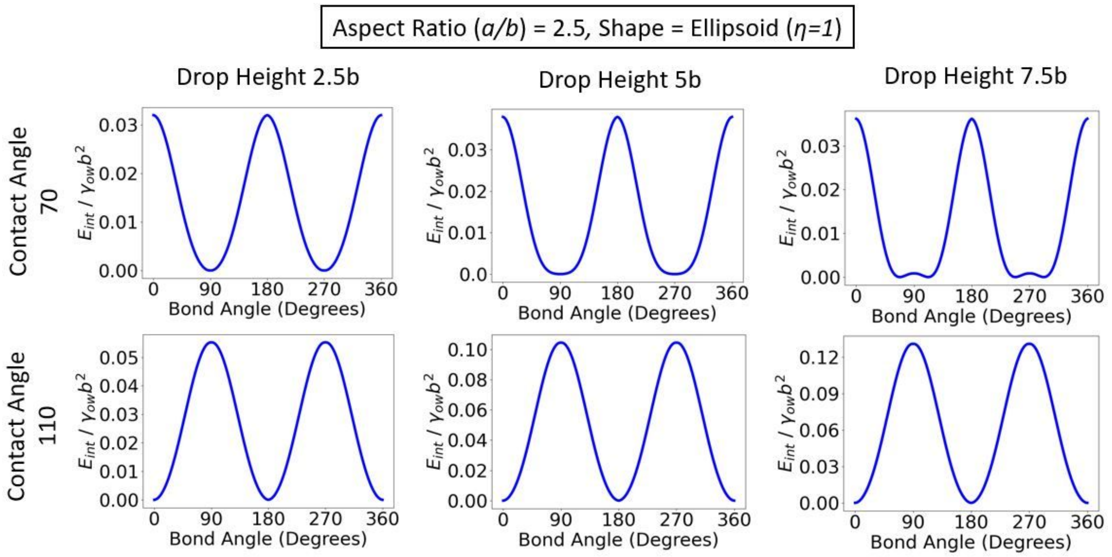

Figure S2. Interfacial energy as a function of bond angle for ellipsoids adsorbed at a cylindrical interface with different contact angles and drop heights at fixed W = 20b. All ellipsoids have $\theta_p = 0°$, $x_p = 0$, $\theta_t = 0°$.

Finally, as discussed in the main paper, ellipsoids adsorbed at a flat interface tend to approach each other tip-to-tip initially then 'roll-over' into the side-to-side configuration but this roll-over transition is suppressed at a cylindrical interface due to interfacial curvature. We can estimate the minimum cylindrical drop height (and hence interfacial curvature) required to suppress the roll-over transition by equating the energy barrier for the roll-over transition ΔE_b to the reduction in capillary energy achieved by this transition. We illustrate this point by considering ellipsoids with aspect ratio $a/b = 2.5$ and contact angle $\theta_w = 110°$, where we have data on the rotational energy landscape for different drop heights (Figure S2, bottom row). Using the data in Figure S2, we can calculate the energy barrier from $\Delta E_b = E_{int}(\theta_b = 90°) - E_{int}(\theta_b = 0°)$ and the radius of curvature of the cylinder from $R = \left(h^2 + w^2/4\right)/2h$. From the leading order multipole expansion of the interfacial energy, we expect $\Delta E_b = A/R$, where A is a prefactor that depends on the characteristics of the capillary quadrupole such as contact line radius and the amplitude of the contact line undulation.[1] By fitting this scaling form to the ΔE_b verses $1/R$ data in Figure S3, we find $A = 1.32\gamma_{ow}b^3$. As discussed in the main paper, we estimate the reduction in the capillary energy achieved by the roll-over transition for this system to be $\approx 0.001\gamma_{ow}b^2$. By equating ΔE_b from the straight-line fit in Figure S3 to $0.001\gamma_{ow}b^2$, we find that the roll-over transition is suppressed for $b/R > 8 \times 10^{-4}$ which corresponds to $h > 0.04b$. This condition is easily satisfied in the flotation regime $h > b$ we are considering in this paper.

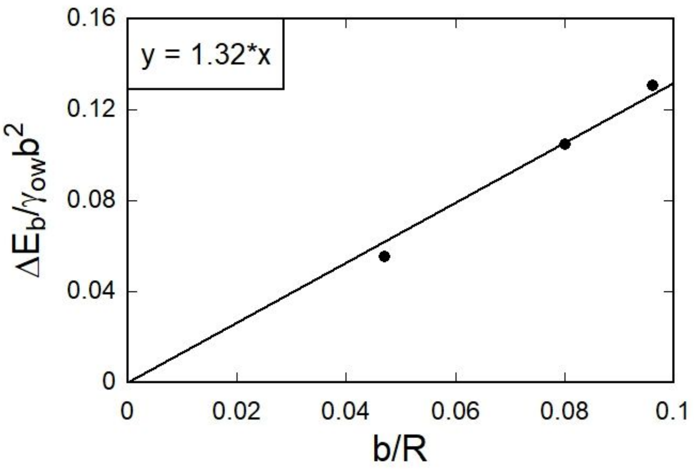

Figure S3. Energy barrier for the roll-over transition ΔE_b *verses interfacial curvature* $1/R$ *for ellipsoids with aspect ratio* $a/b = 2.5$*, contact angle* $\theta_w = 110°$ *adsorbed at a cylindrical interface. The data points are calculated from the data in Figure S2 while the straight line is the best fit of the data to the scaling form* $\Delta E_b = A/R$*.*

References

1. M. Cavallaro, Jr., L. Botto, E. P. Lewandowski, M. Wang, K. J. Stebe, Curvature-driven capillary migration and assembly of rod-like particles, *Proc. Natl. Acad. Sci. U.S.A.*, 2011, **108**, 20923.
